# von Willebrand factor, ADAMTS-13, and thrombospondin 1 in relation to clinical outcomes in elderly patients with a recent myocardial infarction

**DOI:** 10.1016/j.rpth.2023.100164

**Published:** 2023-04-23

**Authors:** Ellen M.K. Warlo, Are A. Kalstad, Peder L. Myhre, Svein Solheim, Harald Arnesen, Arnljot Tveit, Pål Andre Holme, Ingebjørg Seljeflot, Vibeke Bratseth

**Affiliations:** 1 Center for Clinical Heart Research, Department of Cardiology, Oslo University Hospital, Ullevaal, Oslo, Norway; 2 Faculty of Medicine, University of Oslo, Oslo, Norway; 3 Department of Cardiology, Akershus University Hospital, Lørenskog, Norway; 4 Department of Medical Research, Vestre Viken Hospital Trust, Bærum Hospital, Gjettum, Norway; 5 Department of Haematology, Oslo University Hospital, Oslo, Norway

**Keywords:** ADAMTS-13, aged, atrial fibrillation, cardiovascular diseases, thrombospondin 1, von Willebrand factor

## Abstract

**Background:**

von Willebrand factor (VWF) multimers are cleaved by A disintegrin and metalloproteinase with thrombospondin type 1 motif, member 13 (ADAMTS-13) into less active fragments. Thrombospondin 1 (TSP-1) competes with VWF’s cleavage site, protecting it from degradation. Low ADAMTS-13 and high VWF have been associated with cardiovascular disease and atrial fibrillation (AF).

**Objectives:**

We aimed to investigate whether VWF, ADAMTS-13, and TSP-1 are associated with clinical outcome.

**Methods:**

Elderly patients with a recent myocardial infarction (MI) (n = 1027) were followed for 2 years. Blood was collected 2 to 8 weeks after the MI for ADAMTS-13, VWF, and TSP-1 measures. The primary endpoints (major adverse cardiovascular events; n = 210) included the first event of MI, stroke, heart failure hospitalization, coronary revascularization, and all-cause death. Total mortality was also registered (n = 56). The secondary endpoint was new-onset AF (n = 43).

**Results:**

Concentrations of VWF, ADAMTS-13, and TSP-1 did not intercorrelate. The risk of major adverse cardiovascular events was altered in patients with VWF ≥ median (hazard ratio [HR], 1.4; 95% CI, 1.0-1.8; *P* = .03) and ADAMTS-13 ≥ median (HR, 0.7; 95% CI, 0.5-0.9; *P* = .02); however, it was not significant in adjusted models. VWF and ADAMTS-13 were significantly associated with total mortality, with a HR of 2.7 (95% CI, 1.6-4.6; *P* < .001) for VWF (Q4 vs. Q1-Q3) and HR of 0.3 (95% CI, 0.2-0.5; *P* < .001) for ADAMTS-13 (Q2-4 vs. Q1). The associations persisted in multivariable analysis, but the significance disappeared for VWF after correcting for high-sensitivity C-reactive protein. The risk of new-onset AF was lower in patients with VWF ≥ median (HR, 0.5; 95% CI, 0.3-1.0; *P* = .04]), and this was still significant after adjustments.

**Conclusion:**

Although low ADAMTS-13 predicted death, the cardiovascular risk associated with VWF and ADAMTS-13 was weaker than previously reported. Low VWF is associated with new-onset AF and needs further research.

## Essentials

• Thrombospondin 1 (TSP-1) may inhibit ADAMTS-13 cleavage of von Willebrand factor (VWF) multimers.
• Elderly with a recent MI were followed for 2 years for cardiovascular events (CVEs).
• ADAMTS-13, VWF, and TSP-1 did not intercorrelate or predict CVEs after adjusting for covariates.
• Low ADAMTS-13 predicted death, and low VWF was associated with new-onset atrial fibrillation.

## 1 Introduction

von Willebrand factor (VWF) is a marker of endothelial dysfunction and a prothrombotic protein central in primary hemostasis, including platelet adhesion, and secondary hemostasis, with formation of the VWF-factor VIII complex preventing rapid clearance of factor VIII. It exists in different lengths in the circulation, and the largest multimers are most active [1]. VWF multimers are cleaved into smaller and less active fragments by A disintegrin and metalloproteinase with thrombospondin type 1 motif, member 13 (ADAMTS-13). The importance of this interaction has been described in thrombotic thrombocytopenic purpura, where deficiency of ADAMTS-13 leads to accumulation of ultralarge VWF multimers, with subsequent formation of thrombi in small vessels, leading to organ failure and potentially death. Thrombospondin 1 (TSP-1) is a glycoprotein with a wide range of functions [2], one of which is to bind to VWF at the cleavage site, thereby competing with ADAMTS-13 and potentially leading to less cleavage [3].

Cardiovascular disease (CVD) is a major public health problem, and even though treatment strategies have improved considerably, the risk of recurrent events remains high [4]. Elderly patients are especially susceptible and are underrepresented in clinical trials. Increased knowledge of pathophysiologic mechanisms and tools to identify high-risk patients are needed to improve outcome. VWF, ADAMTS-13, and TSP-1 have been associated with CVD. However, the results are diverging and limited in elderly patients [[5], [6], [7]].

Atrial fibrillation (AF) is the most common clinically relevant cardiac arrhythmia, and its prevalence increases with age. The pathogenesis behind AF is unclear, but a mechanistic link between endothelial dysfunction and AF has been discussed [8]. VWF and ADAMTS-13 have been associated with AF, but most studies are cross-sectional or investigating the thrombotic risk in patients with pre-existing AF [9,10]. In the Framingham Heart Study, a prospective cohort, an association between low ADAMTS-13 and incident AF was shown [11]. In contrast, no association between VWF and ADAMTS-13 and new-onset AF was seen in the general population investigated in the Rotterdam study [12]. Reports on TSP-1 in relation to AF are limited, but it has been associated with atrial arrhythmia after myocardial infarction (MI) [13].

In this study on elderly patients with a recent MI, we aimed to investigate the interplay between ADAMTS-13, VWF, and TSP-1 and to assess any associations with major adverse cardiovascular events (MACEs), total mortality, and new-onset AF after 2 years. Our hypotheses were that ADAMTS-13 would be inversely correlated with VWF and TSP-1 and that patients experiencing endpoints would have higher VWF, lower ADAMTS-13, and higher TSP-1 at inclusion.

## 2 Methods

### 2.1 Study design

This was an observational substudy in the OMega-3 fatty acids in Elderly patients with Myocardial Infarction trial [14]. The OMega-3 fatty acids in Elderly patients with Myocardial Infarction trial was a randomized controlled trial including 1027 patients enrolled between 2012 and 2018 at 4 centers in Norway (Oslo University Hospital Ullevål, Akershus University Hospital, Vestre Viken Bærum Hospital, and Stavanger University Hospital). The design of the trial has previously been published [15]. The study was approved by the Regional Committee for Medical and Health Research Ethics (#2012/1422) and conducted in accordance with the declaration of Helsinki and the guidelines for Good Clinical Practice. The trial was registered at https://www.clinicaltrials.gov/ (NCT01841944). All patients gave their written informed consent. The present investigation is based on blood samples at inclusion.

### 2.2 Participants

Patients aged 70 to 82 years were included 2 to 8 weeks after they experienced an MI. Exclusion criteria were participation in other clinical trials, documented intolerance for n-3 fatty acids, and disease states thought to interfere with adherence or reduced life expectancy [15]. Patients were randomized to either 1.8 g of marine omega-3 polyunsaturated fatty acids or corn oil as placebo daily and followed for 2 years. During the inclusion period, 4027 patients were screened, and 1027 were enrolled in the trial. Three thousand patients were not included mostly because they met the exclusion criteria or declined to participate. Additional details regarding the study design and participants have been published [14].

### 2.3 Clinical and outcome data

Clinical data were collected from patient interviews, clinical examinations, and medical records. Diabetes was defined as a previous diagnosis of type 1 or type 2 diabetes mellitus. Patients with a history of AF were classified into AF (before index MI) or AF (at inclusion). AF (at inclusion) involves all patients with AF before, during, and after the MI until inclusion. All variants of AF were registered (paroxysmal, persistent, or permanent). Current smoking was defined as regular tobacco smoking or cessation of smoking 3 months or less prior to inclusion. The primary endpoint (MACE) was the first event of the composite of nonfatal MI, unscheduled revascularization, stroke, hospitalization for heart failure, or all-cause death, ie, death due to any reason. In addition, total mortality of any cause was recorded, where patients who died after the first MACE, were also were included. The secondary outcome was new-onset AF, defined as >30 seconds with no discernible repeating p-waves and irregular RR intervals on electrocardiograms (ECGs) or as documented in clinical records by an internal medicine department [16]. New-onset AF was diagnosed based on access to clinical records and ECGs at study visits (3, 12, and 24 months). In addition, patients without AF at 12 months were invited, of whom 567 accepted to undergo a 2-week intermittent screening with an ambulatory hand-held ECG device (Zenicor Medical Systems AB). Total mortality was retrieved from Statistics Norway. Clinical endpoints were adjudicated by an independent endpoint committee.

### 2.4 Blood sampling and laboratory analyses

Venous blood samples were drawn at inclusion, ie, 2 to 8 weeks after index MI, in a fasting condition and prior to morning medication (8:00-11:00 am). Serum was prepared from whole blood that was allowed to clot and centrifuged within 1 hour at room temperature and 2000 × *g* for 10 minutes. Citrated (0.129 M in 1:10 dilution) and EDTA blood were kept on ice until centrifugation at 4 °C and 3000 × *g* for 20 minutes. All materials were stored at −80 °C until further analysis. Routine analyses were performed using conventional methods.

High-sensitivity C-reactive protein (hsCRP) was measured in serum by enzyme-linked immunosorbent assay (ELISA) (DRG Instruments). VWF antigen (ag), ADAMTS-13 ag, and ADAMTS-13 activity (act) were measured in citrated plasma by use of Asserachrom VWF Ag (Stago Diagnostica), IMUBINDADAMTS-13 ELISA (BioMedica Diagnostics), and TECHNOZYM ADAMTS13 activity ELISA (Technoclone,), respectively. Ratios of VWF/ADAMTS-13 were computed as the combination of high VWF and low ADAMTS-13 is regarded to be particularly harmful [7]. TSP-1 was measured in EDTA plasma by use of Quantikine ELISA Human Thrombospondin-1 (BioTechne). Interassay coefficients of variation in our laboratory were 8.9%, 8.2%, 11.5%, 10.6%, and 8.4%, respectively. hsCRP was successfully analyzed in 1025 patients, VWF in 1022, ADAMTS-13 ag in 1023, ADAMTS-13 act in 1022, and TSP1 in 1019.

### 2.5 Statistical analysis

Analyses on baseline data were performed on all patients included in the study (n = 1027), while outcome data were reported for patients with available follow-up data (n = 1014). Analyses regarding new-onset AF were performed on patients with available follow-up data and no history of AF at inclusion (n = 759). Continuous variables are presented as mean ± SD when normally distributed or median (25th, 75th percentiles) when skewed. Categorical data are presented as numbers or percentages. Student’s unpaired *t*-test and Mann–Whitney U-test were used to compare continuous data with normal or skewed distributions, respectively. Group comparisons for categorical variables were performed by Pearson chi-squared test. Spearman rho was used for correlation analyses. Cox proportional hazard regression was used to assess the association of the biomarkers with outcomes, and results were reported as hazard ratios (HRs) with 95% CIs. The Kaplan-Meier estimator was used to estimate survival curves, and differences in survival distribution were assessed by use of the log-rank test. The proportional hazard assumption test based on Schoenfeld residuals was performed on all cox regression analyses, and a global test with *P* > .05 was accepted. The outcomes were time-to-first occurrence of a MACE, total mortality irrespective of previous nonfatal events, and new-onset AF. In multivariable analyses, age and sex were included by convention. Variables that were associated with both the biomarkers and the endpoints, according to Tables 1 and 2 as well as Supplementary Tables S2 to S4, were considered. Anticoagulation was not included due to its relationship with AF at baseline. The acute phase is known to affect VWF and ADAMTS-13 levels [17], and as blood samples were taken 2 to 8 weeks after MI, this may have affected the results. To limit this, potential bias adjustments for hsCRP were additionally performed. Although the results of the main trial showed no effect of the intervention [14], additional adjustments for the treatment intervention were performed due to randomized principles. A 2-tailed *P* value of <.05 was considered statistically significant. Data were processed using IBM SPSS, versions 26 and 28, and Stata, version 17.

**Table 1:** Baseline characteristics of the total study population at inclusion and according to major adverse cardiovascular events.^a^

Characteristics	Total population (n = 1027)	MACE+ (n = 210)	MACE− (n = 804)
Age, y	74 (72, 78)	75 (72, 78)	74 (72, 77)
Sex (female)	299 (29.1)	48 (22.9)	246 (30.6)
Race (White)	1025 (99.8)	210 (100)	802 (99.8)
Body mass index, kg/m^2^	26.2 (23.9, 28.7)	25.9 (23.7, 28.4)	26.3 (24.0, 29.0)
Cardiovascular risk factors			
Current smoking	124 (12.1)	28 (13.3)	93 (11.6)
Hypertension	620 (60.4)	141 (67.1)	470 (58.5)
Diabetes mellitus	213 (20.7)	68 (32.4)	142 (17.7)
AF before index MI	155 (15.1)	48 (22.9)	106 (13.2)
AF at inclusion	258 (25.1)	75 (35.7)	180 (22.4)
Previous CVD^b^	471 (45.9)	133 (63.3)	334 (41.5)
Previous MI	265 (25.8)	85 (40.5)	176 (21.9)
Previous ischemic stroke	100 (9.7)	33 (15.7)	65 (8.1)
Systolic blood pressure, mmHg	137 ± 20	139 ± 22	137 ± 19
Diastolic blood pressure, mmHg	74 ± 11	72 ± 14	74 ± 11
Biochemical analyses			
Total cholesterol, mmol/L	3.7 ± 0.8	3.6 ± 0.9	3.7 ± 0.8
LDL cholesterol, mmol/L	2.0 ± 0.7	2.0 ± 0.7	2.0 ± 0.7
HDL cholesterol, mmol/L	1.3 ± 0.4	1.2 ± 0.4	1.3 ± 0.4
Triglycerides, mmol/L	1.1 (0.8, 1.5)	1.1 (0.8, 1.5)	1.1 (0.8, 1.5)
hsCRP, mg/L	2.07 (1.07, 3.74)	2.33 (1.14, 4.33)	1.95 (1.03, 3.59)
Medication			
Aspirin	966 (94.1)	197 (93.8)	757 (94.2)
Dual-antiplatelet therapy	882 (85.9)	178 (84.8)	693 (86.2)
Anticoagulation	189 (18.4)	52 (24.8)	134 (16.7)
Statins	991 (96.5)	198 (94.3)	780 (97.0)
β-blockers	853 (83.1)	181 (86.2)	660 (82.1)
Antihypertensives (excluding β-blockers)	736 (71.7)	166 (79.0)	561 (69.8)

AF, atrial fibrillation; CVD, cardiovascular disease; hsCRP, high-sensitivity C-reactive protein; HDL, high-density lipoprotein; LDL, low-density lipoprotein; MACE, major adverse cardiovascular event; MI, myocardial infarction.

a Analyses were performed on all patients included in the study (n = 1027) and those with available follow-up data (n = 1014). Values are given as mean ± SD, median (25th, 75th percentiles) or numbers (%) as appropriate. *P* value of Mann–Whitney U-test, Student’s *t*-test, or chi-squared tests comparing groups with (+) or without (−) major adverse cardiovascular events as appropriate.

b Previous stable angina, unstable angina, MI, percutaneous coronary intervention, coronary artery bypass graft, and ischemic stroke.

**Table 2:** Baseline levels of the markers according to atrial fibrillation at inclusion.^a^

Subgroups		n	VWF (IU/mL)	ADAMTS-13 antigen (ng/mL)	ADAMTS-13 activity (IU/mL)	VWF/ADAMTS-13 antigen (× 10^−3^ IU/ng)	VWF/ADAMTS-13 activity	TSP-1 (ng/mL)
AF before index MI	+	155	1.55 (1.18, 1.92)	693 (609, 809)	0.81 (0.71, 0.93)	2.19 (1.62, 2.77)	1.82 (1.35, 2.38)	123 (68, 365)
	−	872	1.41 (1.09, 1.81)	673 (597, 755)	0.79 (0.69, 0.88)	2.09 (1.59, 2.84)	1.81 (1.34, 2.52)	154 (70, 315)
	*P*		.06	.02^b^	.01^b^	.71	.74	.61
AF at inclusion	+	258	1.57 (1.20, 1.94)	685 (603, 781)	0.80 (0.69, 0.89)	2.23 (1.67, 2.96)	1.96 (1.45, 2.62)	147 (64, 348)
	−	769	1.39 (1.08, 1.77)	675 (602, 756)	0.79 (0.69, 0.90)	2.04 (1.55, 2.78)	1.77 (1.33, 2.42)	150 (70, 310)
	*P*		<.001^b^	.44	.77	.008^b^	.04^b^	.84

ADAMTS-13, A disintegrin and metalloproteinase with thrombospondin type 1 motif, member 13; AF, atrial fibrillation; MI, myocardial infarction; TSP-1, thrombospondin 1; VWF, von Willebrand factor.

a Analyses were performed on all patients included in the study (n = 1027). Values are given as median (25th, 75th percentiles). *P* value of Mann–Whitney U-test, comparing differences in the markers between the groups with (+) or without (−) the clinical entities.

b Significant *P* values.

## 3 Results

Baseline characteristics of the population are shown in Table 1. The total population (n = 1027) was mainly Caucasian, median age was 74 years, and majority were men (71%) and hypertensive (60%). A history of AF before the index MI was registered in 15% of the participants, whereas 25% had AF at inclusion. The median time between the index MI and time of blood sampling was 35 (27, 45) days.

### 3.1 Intercorrelations among VWF, ADAMTS-13, and TSP-1

Statistically significant but weak inverse correlations were found between VWF and ADAMTS-13 ag (*r* = −0.095, *P* = .002) and between VWF and ADAMTS-13 act (*r* = −0.096, *P* = .002). TSP-1 did not correlate to either VWF or ADAMTS-13. ADAMTS-13 ag and act were significantly intercorrelated (*r* = 0.570, *P* < .001). There were no significant correlations between any of the markers and time to blood sampling (data not shown).

### 3.2 VWF, ADAMTS-13, and TSP-1 in association with MACE

After a median follow-up time of 2 years, follow-up data were available for 1014 patients, and 210 primary endpoints were registered. Baseline characteristics according to MACE are shown in Table 1. Patients with MACEs were more likely to be older, male, hypertensive, diabetic, anticoagulated and to have previous disease, including AF and CVD.

VWF, ADAMTS-13, ratios, and TSP-1 levels in the total population and according to endpoints are shown in Table 3. Patients experiencing a MACE had significantly higher VWF (*P* = .01) and lower ADAMTS-13 ag (*P* = .006), and the significance was more pronounced when the VWF/ADAMTS-13 ag ratio was compared (*P* = .002). No significant differences were found for ADAMTS-13 act, VWF/ADAMTS-13 act, or TSP1.

**Table 3:** Baseline levels of the markers in the total population and according to clinical endpoints.^a^

Groups		n	VWF (IU/mL)	ADAMTS-13 antigen (ng/mL)	ADAMTS-13 activity (IU/mL)	VWF/ADAMTS-13 antigen (× 10^−3^ IU/ng)	VWF/ADAMTS-13 activity	TSP-1 (ng/mL)
Total population		1027	1.42 (1.10, 1.83)	678 (602, 759)	0.80 (0.69, 0.89)	2.10 (1.59, 2.83)	1.81 (1.35, 2.46)	150 (69, 321)
Primary endpoint: MACE	+	210	1.53 (1.14, 1.93)	652 (584, 735)	0.79 (0.69, 0.89)	2.24 (1.70, 3.06)	1.86 (1.46, 2.55)	125 (62, 326)
	−	804	1.39 (1.09, 1.81)	681 (607, 764)	0.80 (0.69, 0.89)	2.05 (1.57, 2.73)	1.79 (1.33, 2.43)	157 (72, 318)
	*P*		.01^b^	.006^b^	.72	.002^b^	.10	.22
Secondary endpoint: new-onset AF	+	43	1.25 (0.98, 1.48)	705 (601, 773)	0.82 (0.73, 0.87)	1.81 (1.44, 2.12)	1.55 (1.29, 1.77)	210 (78, 364)
	−	716	1.39 (1.08, 1.78)	672 (604, 755)	0.80 (0.69, 0.89)	2.05 (1.57, 2.79)	1.79 (1.33, 2.44)	145 (70, 302)
	*P*		.008^b^	.30	.53	.009^b^	.005^b^	.20
Total mortality	+	56	1.78 (1.30, 2.24)	593 (531, 700)	0.74 (0.64, 0.86)	2.85 (2.09, 4.17)	2.32 (1.59, 3.34)	125 (78, 351)
	−	958	1.40 (1.09, 1.81)	680 (606, 761)	0.80 (0.69, 0.89)	2.07 (1.57, 2.76)	1.78 (1.34, 2.42)	152 (69, 317)
	*P*		<.001^b^	<.001^b^	.02^b^	<.001^b^	<.001^b^	.91^b^

ADAMTS-13, A disintegrin and metalloproteinase with thrombospondin type 1 motif, member 13; AF, atrial fibrillation; MACE, major adverse cardiovascular event; TSP-1, thrombospondin 1; VWF, von Willebrand factor.

a Analyses were performed on all patients included in the study (n = 1027) and those with available follow-up data (n = 1014). Values are given as median (25th, 75th percentiles). *P* value of Mann–Whitney U-test, comparing differences in the markers between the groups with (+) or without (−) endpoints.

b Significant *P* values.

**Figure 1:**
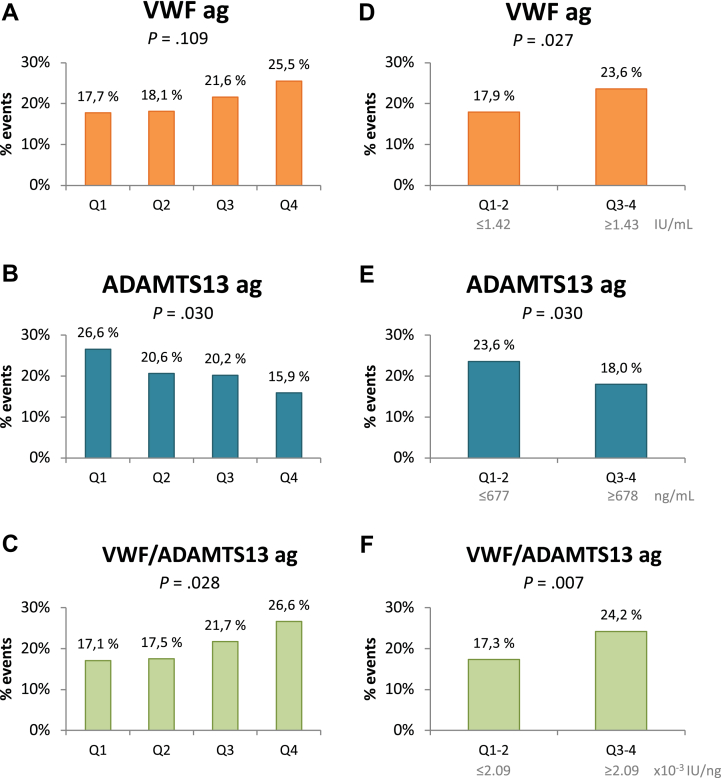
Percentage of major adverse cardiovascular events in the population according to quartiles of (A) VWF, (B) ADAMTS-13 antigen, and (C) VWF/ADAMTS-13 antigen. The *P* values refer to the Pearson chi-squared test. ADAMTS-13, A disintegrin and metalloproteinase with thrombospondin type 1 motif, member 13; ag, antigen; VWF, von Willebrand factor.

To identify any cutoff levels, the biomarkers were divided into quartiles according to the presence of MACE (Figure 1A–C). When dichotomizing at median levels (Figure 1D–F), we found a higher risk for MACE with VWF ≥ median (1.43 IU/mL), with a HR of 1.4 (95% CI, 1.0-1.8; *P* = .03) (Figure 1D), and VWF/ADAMTS-13 ag ≥ median (2.09 × 10^−3^ IU/ng), with a HR of 1.5 (95% CI, 1.1-1.9; *P* = .006) (Figure 1F). Lower risk was observed with ADAMTS-13 ag ≥ median (678 ng/mL), with a HR of 0.7 (95% CI, 0.5-0.9; *P* = .02) (Figure 1E). However, the associations were no longer significant after adjusting for age, sex, previous CVD, AF at inclusion, dual-antiplatelet therapy (DAPT), and hsCRP (Table 4).

**Table 4:** VWF, ADAMTS-13 antigen, and their ratio dichotomized at median level in association to incident major adverse cardiovascular events.^a^

Biomarkers	Univariate analysis		Multivariable analysis Model 1^b^		Multivariable analysis Model 2^c^	
	HR (95% CI)	*P* value	HR (95% CI)	*P* value	HR (95% CI)	*P* value
VWF	1.4 (1.0-1.8)	.03^d^	1.2 (0.9-1.5)	.32	1.1 (0.8-1.5)	.52
ADAMTS-13 ag	0.7 (0.5-0.9)	.02^d^	0.8 (0.6-1.0)	.04^d^	0.8 (0.6-1.0)	.07
VWF/ADAMTS-13 ag	1.5 (1.1-1.9)	.006^d^	1.2 (0.9-1.6)	.16	1.2 (0.9-1.6)	.29

ADAMTS-13, A disintegrin and metalloproteinase with thrombospondin type 1 motif, member 13; Ag, antigen; HR, hazard ratio; VWF, von Willebrand factor.

a HR and *P* values corresponding to the cox regression analysis.

b Adjusted for age, sex, previous cardiovascular disease, atrial fibrillation at inclusion, and dual-antiplatelet therapy.

c Adjusted for age, sex, previous cardiovascular disease, atrial fibrillation at inclusion, dual-antiplatelet therapy, and high-sensitivity C-reactive protein.

d Significant *P* values.

We further analyzed the components of MACE and found that the associations with VWF, ADAMTS-13 ag, and VWF/ADAMTS-13 ag were mainly driven by death (*P* ≤ .001 for all) (Supplementary Table S1).

### 3.3 VWF, ADAMTS-13, and TSP-1 in association with total mortality

When analyzing these markers according to total mortality (n = 56) (Table 3), we found significantly higher VWF, lower ADAMTS-13 ag and act, and higher VWF/ADAMTS-13 ratios in patients who died during the study. No differences were found for TSP-1.

**Figure 2:**
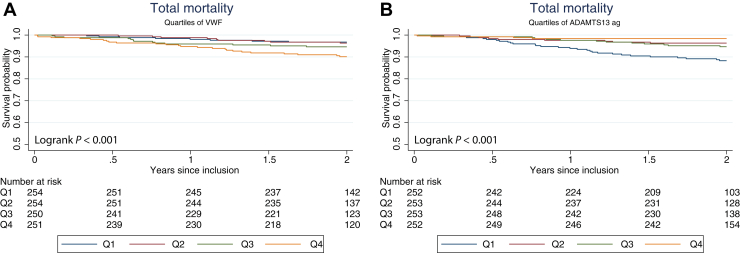
Survival curve for quartiles of (A) VWF antigen and (B) ADAMTS-13 antigen according to total mortality. The *P* value refers to the log-rank test and represents the difference across quartiles. ADAMTS-13, A disintegrin and metalloproteinase with thrombospondin type 1 motif, member 13; ag, antigen; VWF, von Willebrand factor.

**Figure 3:**
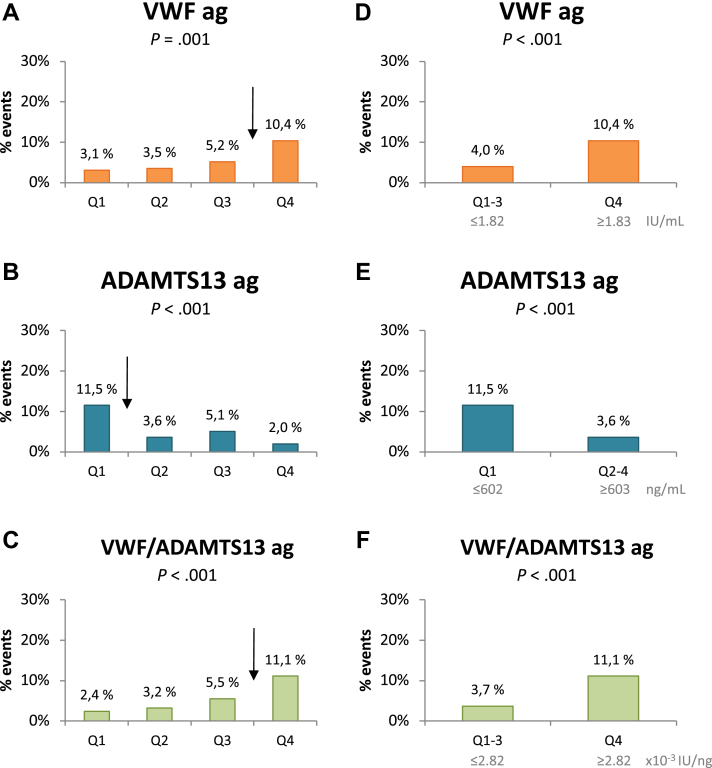
Percentage of total mortality in the population according to quartiles of VWF, ADAMTS-13 antigen, and VWF/ADAMTS-13 antigen. The arrows indicate potential cutoff levels. The *P* values refer to the Pearson chi-squared test. ADAMTS-13, A disintegrin and metalloproteinase with thrombospondin type 1 motif, member 13; ag, antigen; VWF, von Willebrand factor.

The Kaplan-Meier survival curves according to VWF and ADAMTS-13 ag quartiles are shown in Figure 2. We found significant differences across quartiles according to the number of deaths with potential cutoff levels, as indicated in Figure 3, for VWF and VWF/ADAMTS-13 ag between Q1 to Q3 and Q4 and for ADAMTS-13 ag between Q1 and Q2 to Q4. When dichotomized at these levels, a higher event rate was demonstrated in the highest quartile of VWF (≥1.83 IU/mL), with a HR of 2.7 (95% CI, 1.6-4.6; *P* < .001) (Figure 3D), and VWF/ADAMTS-13 ag (≥2.82 × 10^−3^ IU/ng), with a HR of 3.2 (95% CI, 1.9-5.3; *P* < .001) (Figure 3F). ADAMTS-13 ag levels in the higher quartiles (Q2-Q4) (≥603 ng/mL), compared to those in Q1, showed a HR of 0.3 (95% CI, 0.2-0.5; *P* < .001) (Figure 3E). These associations were still significant after adjusting for age, sex, previous CVD, AF at inclusion, and DAPT but not significant for VWF and VWF/ADAMTS-13 ag after adjusting for hsCRP (Table 5). Additional adjustments for the trial intervention groups did not influence the results (data not shown).

**Table 5:** von Willebrand factor, ADAMTS-13 antigen and activity, and the VWF/ADAMTS-13 ratios dichotomized at the specified level in association with total mortality.^a^

Biomarkers	Univariate analysis		Multivariable analysis Model 1^b^		Multivariable analysis Model 2^c^	
	HR (95% CI)	*P* value	HR (95% CI)	*P* value	HR (95% CI)	*P* value
VWF (Q4 vs Q1-3)	2.7 (1.6-4.6)	<.001^d^	2.1 (1.2-3.5)	.007^d^	1.6 (0.9-2.8)	.11
ADAMTS-13 ag (Q2-4 vs Q1)	0.3 (0.2-0.5)	<.001^d^	0.4 (0.2-0.6)	<.001^d^	0.4 (0.2-0.6)	<.001^d^
ADAMTS-13 act (Q2-4 vs Q1)	0.5 (0.3-0.9)	.02^d^	0.6 (0.3-1.0)	.05^d^	0.7 (0.4-1.1)	.13
VWF/ADAMTS-13 ag (Q4 vs Q1-3)	3.2 (1.9-5.3)	<.001^d^	2.3 (1.3-3.9)	.003^d^	1.8 (1.0-3.1)	.05
VWF/ADAMTS-13 act (Q4 vs Q1-3)	2.2 (1.3-3.7)	.004^d^	1.8 (1.0-3.0)	.04^d^	1.5 (0.8-2.6)	.17

ADAMTS-13, A disintegrin and metalloproteinase with thrombospondin type 1 motif, member 13; ag, antigen; act, activity; HR, hazard ratio; VWF, von Willebrand factor.

a HR and *P* values corresponding to the cox regression analysis.

b Adjusted age, sex, previous cardiovascular disease, atrial fibrillation at inclusion, and dual-antiplatelet therapy.

c Adjusted for age, sex, previous cardiovascular disease, atrial fibrillation at inclusion, dual-antiplatelet therapy, and high-sensitivity C-reactive protein.

d Significant *P* values.

### 3.4 VWF, ADAMTS-13, and TSP1 in association with AF

VWF, ADAMTS-13, and TSP1 levels in patients with and without AF at baseline are presented in Table 2. The number of patients with a history of AF before the index MI was 155 and increased to 258 at inclusion. Patients with AF before MI had significantly higher ADAMTS-13 ag and act levels (*P* = .02 and *P* = .01, respectively) and borderline significantly higher VWF (*P* = .06) at baseline. When investigating the patients with AF at inclusion, the difference in VWF was more pronounced (*P* < .001), while the difference in ADAMTS-13 was no longer significant. No associations between TSP-1 and AF were observed.

The baseline characteristics of the 759 patients with available follow-up data and no history of AF at inclusion are shown in Supplementary Table S4. After 2 years, new-onset AF was registered in 43 patients. Patient characteristics were similar between patients with new-onset AF and those without new-onset AF (Supplementary Table S4). Levels of VWF, ADAMTS-13, ratios, and TSP-1 according to new-onset AF are presented in Table 3. Patients with new-onset AF had significantly lower levels of VWF and VWF/ADAMTS-13 ratios, numerically lower and higher ADAMTS-13 ag and TSP-1 levels, respectively. We found significantly lower risks of new-onset AF in patients with VWF ≥ median (1.39 IU/mL), with a HR of 0.5 (95% CI, 0.3-1.0; *P* = .04); VWF/ADAMTS-13 ag ≥ median (2.04 × 10^−3^ IU/ng), with a HR of 0.5 (95% CI, 0.3-1.0; *P* = .05); and VWF/ADAMTS-13 act ≥ median (1.76), with a HR of 0.4 (95% CI, 0.2-0.7; *P* = .004). Additional adjustments for age, sex, hsCRP, or the randomized treatment principle did not influence the results (data not shown).

## 4 Discussion

In our elderly patients with a recent MI, the main findings were that VWF and ADAMTS-13 were associated with MACEs in univariate analyses but not significant after adjusting for covariates. Low ADAMTS-13 and high VWF levels predicted total mortality, but for VWF, the significance was lost when correcting for inflammation. Patients with lower VWF and VWF/ADAMTS-13 levels had significantly higher risk for developing new-onset AF during follow-up, whereas patients with AF at inclusion had higher VWF and VWF/ADAMTS-13. TSP-1 was not associated with any clinical entity at baseline or any of the endpoints.

### 4.1 Correlations

We found VWF and ADAMTS-13 to be significantly but weakly correlated, in accordance with other studies showing weak or no correlations [18,19]. In our population, TSP-1 did not correlate to either VWF or ADAMTS-13, in contrast to our hypothesis. Comparable studies are limited, but there are 2 articles reporting TSP-1 to be inversely correlated with ADAMTS-13 in patients with acute coronary syndrome and adults with sickle cell disease but not in healthy controls [20,21]. In addition, a positive association between TSP1 and VWF was reported in healthy controls but not in patients with sickle cell disease [21].

The lack of correlations may be due to the choice of method as only VWF ag and not VWF act was measured; however, these methods seem to intercorrelate [22,23]. It is also important to emphasize that the interactions between these markers are complex. VWF needs to be unfolded to be accessible for cleavage by ADAMTS-13, and this especially occurs in vessels with high shear stress [1]. It may be that the mechanistic forces *in vivo* are more important for the synergy of these proteins than the circulating levels measured *ex vivo*. Also, these markers, and especially TSP-1, have other properties beyond the VWF-ADAMTS-13 axis, which may influence blood levels [1,2]. Almost all patients were on DAPT, which may reduce the release of VWF and TSP1 from platelets.

### 4.2 MACEs

Patients experiencing a MACE had significantly higher VWF, lower ADAMTS-13 ag, and higher VWF/ADAMTS-13 ag at baseline. Nevertheless, this was not significant in the multivariable analysis. Reports on VWF and ADAMTS-13 concentrations in association with CVD are diverging. VWF has repeatedly been associated with coronary heart disease [24]; however, it has been suggested that the associations are more modest than previously reported [25]. Associations between ADAMTS-13 and CVD have also been shown in previous studies [6,26]. A meta-analysis found no significant association between ADAMTS-13 and coronary heart disease, which may be due to wide CIs, suggesting lack of power [7].

The differences in risk estimates in previous reports may be due to not only differences in study populations and endpoint definitions but also the choice of covariates included in multivariable analysis, such as inflammatory markers like C-reactive protein (CRP) [7]. The acute phase response has been shown to increase VWF and lower ADAMTS-13 levels [17]. Our results indicate that the association between these markers and MACE is of less importance in this particular population with high age and established CVD.

We found no difference in TSP-1 levels in patients developing a MACE. High TSP-1 has been associated with increased risk of cardiovascular events in patients with acute coronary syndrome and ischemic stroke [20,27]; however, the literature is limited. Varying results may be due to timing of the blood sampling as TSP-1 levels have been shown to rapidly decline after percutaneous coronary intervention, probably due to antiplatelet therapy [28]. False elevation of TSP-1 concentrations is also a potential problem due to the challenge with platelet activation and degranulation *ex vivo* [27].

### 4.3 Total mortality

Higher VWF and VWF/ADAMTS-13 ratios and lower ADAMTS-13 ag and act were associated with an increased risk of total mortality. In models adjusting for CRP, the association was no longer significant for VWF, ADAMTS-13 act, and VWF/ADAMTS-13 ratios, while ADAMTS-13 ag remained significant. This is partly in accordance with previous results.

In the Rotterdam study, a population-based cohort, higher VWF and lower ADAMTS-13 act were associated with increased risk of all-cause death [29]. The results remained significant after adjustments but not adjusted for CRP, similarly reported by others [30,31]. There are also reports on loss of significance after adjustments for traditional cardiovascular risk factors and CRP [32,33]. On the contrary, some have shown the association to persist after adjustments, including acute phase responses [34].

Nevertheless, variations in age and diseases make comparisons challenging. Our results indicate that the VWF-ADAMTS-13 axis, and especially ADAMTS-13 ag, are associated with the risk of mortality in elderly high-risk patients. Even though the significance is lost for VWF after adjusting for hsCRP, it does not necessarily rule out VWF’s role in the pathogenesis. The importance of VWF as an initiator of thrombosis is well established, and as it is known that VWF increases during inflammation, we hypothesize that the association between VWF and death may partly be driven by an inflammatory pathway. The concept of “immunothrombosis” has been introduced, and it is suggested that inflammation activates thrombosis due to an imbalance between VWF and ADAMTS-13. VWF has been suggested to work as a bridge between thrombosis and inflammation through its interactions with platelets and leukocytes [1,35]. In our population, ADAMTS-13 seems to be less dependent on hsCRP levels compared to VWF. This is in accordance with a previous report in a healthy young population showing weaker associations between ADAMTS-13 and CRP than between VWF and CRP [36].

In this population, ADAMTS-13 ag seems to be a better marker of disease risk compared to ADAMTS-13 act. This is not easily understood as one would expect that the function of the enzyme is the most important. These 2 methods correlate moderately; thus, they may partly reflect different features of ADAMTS-13. It may also be that activity measurement is more prone to other interfering variables. For instance, ADAMTS-13 act may be reduced during storage and incubation [37]. In addition, the ADAMTS-13 act analysis seems not to reflect the more complex interaction with VWF under shear stress *in vivo* [37].

We found no associations between TSP-1 and total mortality, in line with previous research [34]. On the other hand, high TSP-1 has been associated with mortality after ischemic stroke [27], but again, the literature is limited.

### 4.4 AF

Our elderly patients with new-onset AF had significantly lower VWF at baseline in contrast to our hypothesis. The lack of differences in ADAMTS-13 and TSP-1 is in contrast to reports in younger populations. The Framingham Heart Study (mean age, 55 years) and the Atherosclerosis Risk In Communities study (mean age, 53-57 years) showed that lower ADAMTS-13 and higher VWF were significantly associated with increased risk of incident AF, respectively [11,38]. However, the Rotterdam study (mean age, 69 years) reported no differences in VWF or ADAMTS-13 act [12]. Other studies are mainly cross-sectional and show higher VWF and lower ADAMTS-13 levels in patients with pre-existing AF [9,39]. Nevertheless, it is worth noting that the latter study [39] observed no difference in patients with age ≥75 years, in accordance with a case-control study in elderly patients (mean age, 78 years) that reported no difference in VWF levels with regard to AF [40]. The observation of lower VWF in patients developing AF in our study is unexpected and needs to be further investigated to establish whether this is specific to our population or a general finding.

Cross-sectional analysis of our baseline data regarding AF shows somewhat diverging results. Patients with AF before the index MI had a tendency of higher VWF and significantly higher ADAMTS-13. Furthermore, when investigating all patients with AF at inclusion, including those with post-MI AF, which may reflect another pathophysiologic process, the difference in VWF levels was significant, but the significance for ADAMTS-13 was lost. It should be noted that the numerical difference in VWF levels was relatively small. Although our population is elderly and with established CVD, it still seems possible to detect a modest difference in VWF levels in patients with pre-existing AF. Nevertheless, our results indicate that high VWF and low ADAMTS-13 levels are no longer predictive of new-onset AF at this age. This corresponds with the suggestion that endothelial dysfunction is more predictive of AF in younger patients [8,41].

TSP-1 has previously been weakly associated with the occurrence of atrial arrhythmia after acute MI [13]. No differences were found in our study, indicating no predictive value of TSP-1 with regard to AF in the elderly.

### 4.4 Limitations

This is a substudy of a trial designed for a different purpose; therefore, all analyses are post hoc and should be interpreted cautiously. Preanalytic power analyses were not conducted, and statistical error type II cannot be ruled out. We tested 4 different biomarkers in association with multiple predefined outcomes, but we did not account for multiple testing. The use of quartiles as cutoffs is arbitrary and does not necessarily represent biologically relevant thresholds. The ABO blood type system has been shown to affect VWF levels [36]; unfortunately, blood type was not available in this study. VWF ag was measured and not VWF act; however, these have been shown to correlate [22,23]. The biomarkers were only measured once after MI and may not reflect levels in a stable situation; therefore, adjustments for hsCRP were performed to limit a potential acute phase effect. The investigations were performed in elderly patients with comorbidities, and most of them were treated with DAPT, which may have influenced the results. Nevertheless, we consider the age of the population as a strength of the current study because elderly are underrepresented in the literature.

## 5 Conclusions

In the current elderly population with a recent MI, the association between VWF, ADAMTS-13, and incident MACE seems to be of less importance. TSP1 levels did not predict MACE, total mortality, or new-onset AF, and did not correlate to levels of VWF and ADAMTS-13. The associations between high VWF and VWF/ADAMTS-13 and the presence of AF at inclusion, were not reflected in any predictive value of new-onset AF in our elderly patients. Low ADAMTS-13 levels predicted total mortality, whereas VWF as a predictor of mortality was lost when correcting for inflammation. Thus, we hypothesize that high VWF may be related to mortality through an inflammatory pathway.

## Supplementary material

Supplementary Table S1 Baseline levels of the measured markers according to the primary endpoint components Supplementary Table S2. Baseline characteristics in patients according to total mortality Supplementary Table S3. Baseline levels of the markers according to different clinical entities at inclusion Supplementary Table S4. Baseline characteristics in patients with no history of atrial fibrillation and according to new-onset AF
