# Supplementary material for: von Willebrand factor, ADAMTS-13, and thrombospondin 1 in relation to clinical outcomes in elderly patients with a recent myocardial infarction
Source: Res Pract Thromb Haemost. 2023 Apr 23;7(4):100164. doi: 10.1016/j.rpth.2023.100164 (PMC10225923; doi:10.1016/j.rpth.2023.100164)
Supplement: Supplementary Table S1 — Baseline levels of the measured markers according to the primary endpoint components Supplementary Table S2. Baseline characteristics in patients according to total mortality Supplementary Table S3. Baseline levels of the markers according to different clinical entities at inclusion Supplementary Table S4. Baseline characteristics in patients with no history of atrial fibrillation and according to new-onset AF [file mmc1.docx]

**Supplementary Table S1** Baseline levels of the measured markers according to the primary endpoint components

|  |  | n | VWF (IU/mL) | ADAMTS13 antigen (ng/mL) | ADAMTS13 activity (IU/mL) | VWF/ADAMTS13 antigen (*10^-3^ IU/ng) | VWF/ADAMTS13 activity | TSP1 (ng/mL) |
| --- | --- | --- | --- | --- | --- | --- | --- | --- |
| Death as a first event | + | 38 | 1.92 (1.38, 2.31) | 586 (504, 707) | 0.73 (0.61, 0.86) | 2.93 (2.10, 4.52) | 2.32 (1.83, 3.58) | 113 (71, 246) |
|  | - | 976 | 1.40 (1.09, 1.81) | 679 (605, 761) | 0.80 (0.69, 0.89) | 2.08 (1.57, 2.78) | 1.78 (1.34, 2.43) | 154 (69, 319) |
|  | *p* |  | **<0.001** | **<0.001** | **0.04** | **<0.001** | **<0.001** | 0.33 |
| Acute MI | + | 74 | 1.58 (1.13, 1.97) | 672 (596, 779) | 0.83 (0.70, 0.90) | 2.23 (1.54, 2.97) | 1.81 (1.29, 2.61) | 127 (56, 286) |
|  | - | 940 | 1.41 (1.10, 1.82) | 678 (604, 757) | 0.79 (0.69, 0.89) | 2.09 (1.59, 2.78) | 1.80 (1.35, 2.44) | 153 (71, 323) |
|  | *p* |  | 0.19 | 0.94 | 0.24 | 0.37 | 0.67 | 0.18 |
| Stroke | + | 29 | 1.48 (1.19, 1.78) | 644 (593, 715) | 0.77 (0.69, 0.90) | 2.22 (1.74, 2.78) | 2.02 (1.43, 2.42) | 153 (70, 319) |
|  | - | 985 | 1.42 (1.10, 1.82) | 678 (603, 760) | 0.80 (0.69, 0.89) | 2.09 (1.59, 2.82) | 1.80 (1.34, 2.45) | 91 (45, 191) |
|  | *p* |  | 0.80 | 0.20 | 0.95 | 0.53 | 0.96 | 0.06 |
| Unscheduled revascularization | + | 35 | 1.31 (1.05, 1.50) | 692 (630, 772) | 0.79 (0.70, 0.85) | 1.82 (1.45, 2.19) | 1.64 (1.34, 1.76) | 198 (75, 479) |
|  | - | 979 | 1.43 (1.10, 1.84) | 677 (599, 758) | 0.80 (0.69, 0.89) | 2.11 (1.59, 2.84) | 1.83 (1.34, 2.46) | 147 (69, 315) |
|  | *p* |  | **0.05** | 0.28 | 0.56 | **0.02** | 0.09 | 0.12 |
| Heart failure hospitalization | + | 34 | 1.65 (1.16, 1.88) | 627 (559, 691) | 0.79 (0.69, 0.89) | 2.43 (1.98, 3.36) | 2.08 (1.63, 2.53) | 149 (86, 310) |
|  | - | 980 | 1.41 (1.10, 1.82) | 679 (604, 761) | 0.80 (0.69, 0.89) | 2.08 (1.58, 2.79) | 1.79 (1.34, 2.45) | 149 (69, 320) |
|  | *p* |  | 0.18 | **0.009** | 0.87 | **0.02** | 0.27 | 0.68 |

Analyses are performed on patients with available follow-up data (n=1014). Values are given as median (25^th^, 75^th^ percentiles). *P* value of Mann-Whitney U test, comparing differences in the markers between the groups with (+) or without (-) the endpoints. Significant *p* values are highlighted with boldface. MI: myocardial infarction

**Supplementary Table S2** Baseline characteristics in patients according to total mortality

|  | **Total mortality + (n=56)** | **Total mortality - (n=958)** | **p-value** |
| --- | --- | --- | --- |
| Age, years | 76 (73, 80) | 74 (72, 77) | **<0.001** |
| Sex, female | 10 (17.9) | 284 (29.6) | 0.06 |
| Race, white | 56 (100) | 956 (99.8) | 0.73 |
| Body mass index, kg/m^2^ | 25.9 (22.8, 28.4) | 26.2 (23.9, 28.7) | 0.48 |
| *Cardiovascular risk factors* |  |  |  |
| Current smoking | 9 (16.1) | 112 (11.7) | 0.33 |
| Hypertension | 39 (69.6) | 572 (59.7) | 0.14 |
| Diabetes mellitus | 23 (41.1) | 187 (19.5) | **<0.001** |
| AF before index MI | 17 (30.4) | 137 (14.3) | **0.001** |
| AF at inclusion | 26 (46.4) | 229 (23.9) | **<0.001** |
| Previous CVD^a^ | 38 (67.9) | 429 (44.8) | **<0.001** |
| Previous MI | 25 (44.6) | 236 (24.6) | **<0.001** |
| Previous ischemic stroke | 8 (14.3) | 90 (9.4) | 0.23 |
| Systolic blood pressure, mmHg | 136±24 | 137±19 | 0.55 |
| Diastolic blood pressure, mmHg | 74±20 | 74±11 | 0.97 |
| *Biochemical analyses* |  |  |  |
| Total cholesterol, mmol/L | 3.8±1.1 | 3.7±0.8 | 0.16 |
| LDL cholesterol, mmol/L | 2.1±0.9 | 2.0±0.7 | 0.18 |
| HDL cholesterol, mmol/L | 1.3±0.5 | 1.3±0.4 | 0.99 |
| Triglycerides, mmol/L | 1.1 (0.9, 1.4) | 1.1 (0.8, 1.5) | 0.97 |
| hsCRP | 2.97 (1.32, 6.58) | 1.97 (1.05, 3.65) | **0.002** |
| *Medication* |  |  |  |
| Aspirin | 51 (91.1) | 903 (94.3) | 0.33 |
| Dual antiplatelet therapy | 42 (75.0) | 829 (86.5) | **0.02** |
| Anticoagulation | 21 (37.5) | 165 (17.2) | **<0.001** |
| Statins | 50 (89.3) | 928 (96.9) | **0.003** |
| β-blockers | 50 (89.3) | 791 (82.6) | 0.19 |
| Antihypertensives (excluding β-blockers) | 45 (80.4) | 682 (71.2) | 0.14 |

Analyses are performed on patients with available follow-up data (n=1014). Values are given as mean±SD, median (25th, 75th percentiles) or numbers (%) as appropriate. *P* value of Mann-Whitney U test, Student t-test or chi-squared tests comparing groups with (+) or without (-) total mortality as appropriate. Significant *p* values are highlighted with boldface. AF: Atrial fibrillation; CVD: Cardiovascular disease; MI: myocardial infarction; hsCRP: high sensitivity C-reactive protein.
^a^ Previous stable angina, unstable angina, MI, percutaneous coronary intervention, coronary artery bypass graft and ischemic stroke.

**Supplementary Table S3** Baseline levels of the markers according to different clinical entities at inclusion

|  |  | n | VWF (IU/mL) | ADAMTS13 antigen (ng/mL) | ADAMTS13 activity (IU/mL) | VWF/ADAMTS13 antigen (*10^-3^ IU/ng) | VWF/ADAMTS13 activity | TSP1 (ng/mL) |
| --- | --- | --- | --- | --- | --- | --- | --- | --- |
| Age > median  (74 years) | + | 486 | 1.50 (1.16, 1.89) | 660 (583, 745) | 0.79 (0.68, 0.88) | 2.23 (1.73, 2.97) | 1.93 (1.45, 2.57) | 153 (70, 315) |
|  | - | 541 | 1.36 (1.07, 1.77) | 689 (614, 774) | 0.80 (0.70, 0.89) | 1.99 (1.52, 2.67) | 1.72 (1.29, 2.38) | 147 (69, 323) |
|  | *p* |  | **<0.001** | **<0.001** | 0.16 | **<0.001** | **<0.001** | 0.96 |
| Sex (female) | + | 299 | 1.41 (1.08, 1.80) | 692 (626, 783) | 0.81 (0.70, 0.91) | 2.06 (1.58, 2.54) | 1.76 (1.32, 2.42) | 144 (70, 326) |
|  | - | 728 | 1.43 (1.11, 1.84) | 665 (591, 752) | 0.79 (0.68, 0.88) | 2.11 (1.59, 2.88) | 1.85 (1.36, 2.50) | 153 (69, 316) |
|  | *p* |  | 0.53 | **<0.001** | **0.01** | 0.06 | 0.23 | 0.96 |
| Diabetes | + | 213 | 1.50 (1.15, 1.88) | 685 (607, 768) | 0.80 (0.70, 0.90) | 2.20 (1.62, 2.77) | 1.90 (1.33, 2.51) | 124 (65, 315) |
|  | - | 814 | 1.39 (1.08, 1.82) | 674 (601, 757) | 0.79 (0.69, 0.89) | 2.07 (1.59, 2.83) | 1.77 (1.35, 2.46) | 155 (70, 323) |
|  | *p* |  | 0.07 | 0.32 | 0.40 | 0.31 | 0.39 | 0.25 |
| Hypertension | + | 620 | 1.44 (1.11, 1.90) | 672 (600, 760) | 0.80 (0.70, 0.89) | 2.14 (1.57, 2.90) | 1.81 (1.34, 2.53) | 142 (69, 317) |
|  | - | 407 | 1.39 (1.09, 1.72) | 686 (605, 757) | 0.80 (0.67, 0.89) | 2.01 (1.62, 2.68) | 1.79 (1.35, 2.41) | 159 (70, 323) |
|  | *p* |  | 0.07 | 0.56 | 0.35 | 0.08 | 0.64 | 0.60 |
| Smokers | + | 124 | 1.41 (1.05, 1.78) | 682 (574, 757) | 0.78 (0.70, 0.91) | 2.05 (1.51, 2.82) | 1.71 (1.27, 2.34) | 141 (76, 326) |
|  | - | 903 | 1.43 (1.11, 1.84) | 677 (602, 759) | 0.80 (0.69, 0.89) | 2.10 (1.60, 2.83) | 1.82 (1.36, 2.50) | 152 (68, 318) |
|  | *p* |  | 0.47 | 0.92 | 0.35 | 0.68 | 0.20 | 0.59 |
| Previous CVD^a^ | + | 471 | 1.52 (1.16, 1.95) | 670 (590, 757) | 0.79 (0.69, 0.89) | 2.25 (1.70, 3.04) | 1.94 (1.41, 2.70) | 141 (65, 307) |
|  | - | 556 | 1.34 (1.07, 1.71) | 679 (610, 760) | 0.80 (0.69, 0.89) | 1.98 (1.53, 2.58) | 1.72 (1.31, 2.29) | 157 (71, 324) |
|  | *p* |  | **<0.001** | 0.18 | 0.32 | **<0.001** | **<0.001** | 0.42 |

Analyses are performed on all patients included in the study (n=1027). Values are given as median (25^th^, 75^th^ percentiles). *P* value of Mann-Whitney U test, comparing differences in the markers between the groups with (+) or without (-) the clinical entities. Significant *p* values are highlighted with boldface. MI: myocardial infarction. CVD: Cardiovascular disease. ^a^ Previous stable angina, unstable angina, MI, percutaneous coronary intervention, coronary artery bypass graft and ischemic stroke.

**Supplementary Table S4** Baseline characteristics in patients with no history of atrial fibrillation and according to new-onset AF

|  | **Patients without AF (n=759)** | **New-onset AF + (n=43)** | **New-onset AF - (n=716)** | **p-value** |
| --- | --- | --- | --- | --- |
| Age, years | 74 (72, 78) | 75 (72, 79) | 74 (72, 77) | 0.59 |
| Sex, female | 217 (28.6) | 12 (27.9) | 205 (28.6) | 0.92 |
| Race, white | 757 (99.7) | 43 (100) | 714 (99.7) | 0.73 |
| Body mass index, kg/m^2^ | 26.0 (23.9, 28.7) | 25.6 (24.1, 29.9) | 26.0 (23.9, 28.7) | 0.96 |
| *Cardiovascular risk factors* |  |  |  |  |
| Current smoking | 103 (13.6) | 2 (4.7) | 101 (14.1) | 0.08 |
| Hypertension | 442 (58.2) | 25 (58.1) | 417 (58.2) | 0.99 |
| Diabetes mellitus | 153 (20.2) | 11 (25.6) | 142 (19.8) | 0.36 |
| AF at inclusion | 0 (0) | 0 (0) | 0 (0) | **-** |
| Previous CVD^a^ | 334 (44.0) | 20 (46.5) | 314 (43.9) | 0.73 |
| Previous MI | 190 (25.0) | 11 (25.6) | 179 (25.0) | 0.93 |
| Previous ischemic stroke | 68 (9.0) | 6 (14) | 62 (8.7) | 0.24 |
| Systolic blood pressure, mmHg | 138±20 | 136±22 | 138±20 | 0.60 |
| Diastolic blood pressure, mmHg | 74±11 | 71±10 | 74±11 | 0.07 |
| *Biochemical analyses* |  |  |  |  |
| Total cholesterol, mmol/L | 3.7±0.8 | 3.8±0.8 | 3.6±0.8 | 0.19 |
| LDL cholesterol, mmol/L | 1.9±0.6 | 2.1±0.6 | 1.9±0.6 | 0.07 |
| HDL cholesterol, mmol/L | 1.3±0.4 | 1.3±0.4 | 1.3±0.4 | 0.56 |
| Triglycerides, mmol/L | 1.1 (0.8, 1.5) | 1.02 (0.73, 1.26) | 1.10 (0.85, 1.52) | 0.08 |
| hsCRP, mg/L | 1.91 (1.02, 3.60) | 2.33 (1.15, 3.84) | 1.90 (1.02, 3.57) | 0.42 |
| *Medication* |  |  |  |  |
| Aspirin | 742 (97.8) | 42 (97.7) | 700 (97.8) | 0.97 |
| Dual antiplatelet therapy | 702 (92.5) | 41 (95.3) | 661 (92.3) | 0.46 |
| Anticoagulation | 26 (3.4) | 0 (0) | 26 (3.6) | 0.20 |
| Statins | 744 (98.0) | 43 (100) | 701 (97.9) | 0.34 |
| β-blockers | 614 (80.9) | 36 (83.7) | 578 (80.7) | 0.63 |
| Antihypertensives (excluding β-blockers) | 532 (70.1) | 30 (69.8) | 502 (70.1) | 0.96 |

Analyses are performed on patients with available follow-up data (n=1014). Values are given as mean±SD, median (25th, 75th percentiles) or numbers (%) as appropriate. *P* value of Mann-Whitney U test, Student t-test or chi-squared tests comparing groups with (+) or without (-) new-onset AF as appropriate. Significant *p* values are highlighted with boldface. AF: Atrial fibrillation; CVD: Cardiovascular disease; MI: myocardial infarction; hsCRP: high sensitivity C-reactive protein.
^a^ Previous stable angina, unstable angina, MI, percutaneous coronary intervention, coronary artery bypass graft and ischemic stroke
